# Postoperative Outcomes After Robotic Liver Resection of Caudate Lobe: A Systematic Review

**DOI:** 10.3390/medicina61010034

**Published:** 2024-12-29

**Authors:** Gabriela Del Angel Millan, Gianluca Cassese, Fabio Giannone, Celeste Del Basso, Mariantonietta Alagia, Marco Lodin, Igor Monsellato, Marco Palucci, Federico Sangiuolo, Fabrizio Panaro

**Affiliations:** 1 Division of Hepato-Pancreato-Biliary, Oncologic and Robotic Surgery, Azienda Ospedaliero-Universitaria SS. Antonio e Biagio e Cesare Arrigo, 15121 Alessandria, Italy; 2 HPB and Robotic Surgery Research Unit, Department of Research and Innovation (DAIRI), Azienda Ospedaliero-Universitaria SS. Antonio e Biagio e Cesare Arrigo, 15121 Alessandria, Italy; 3 Department of Health Sciences, School of Medicine, University of Eastern Piedmont “Amedeo Avogadro”, 15121 Alessandria, Italy

**Keywords:** robotic liver resection, robotic caudatectomy, caudate lobectomy

## Abstract

*Background and Objectives*: Resection of the caudate lobe of the liver is considered a highly challenging surgical procedure due to the deep anatomic location of this segment and the relationships with major vessels. There is no clear evidence about the safety and effectiveness of robotic resection of the caudate lobe. The aim of this systematic review was to report data about the safety, technical feasibility, and postoperative outcomes of robotic caudate lobectomy. *Materials and Methods*: A systematic review of the MEDLINE and SCOPUS databases was undertaken, including studies published until 19 December 2024. *Results*: A total of 5 studies including 110 patients were selected. Of these surgeries, 56.3% were performed for malignant tumors. Tumor size varied significantly between 0.9 and 7.7 cm in the largest diameter. The mean operative time was 184.5 min (range 70–522 min), and the estimated blood loss was 95.5 mL (range 10–1500 mL). The median hospital length of stay was 4.2 days (range 2–19 days) and no cases of conversion to open were reported. All the patients underwent R0 resection. In total, 24 out of 110 patients (21.8%) developed postoperative complications, with 1.8% of all patients developing a major complication (Clavien–Dindo classification ≥ III). No perioperative deaths were reported by the included studies. *Conclusions*: Few retrospective studies investigating the outcomes of robotic resection of the caudate lobe are currently available in the literature. From published data, it may be a safe and feasible alternative to open and laparoscopic caudate lobectomy in selected patients in referral HPB centers. Further studies with larger sample sizes are needed to confirm such preliminary findings.

## 1. Introduction

Since the introduction of laparoscopic liver surgery in 1991, its spread has been slower than other areas of surgery. The main concerns were the technical complexity of parenchymal transection and hilar dissection, the oncological concerns about resection margins, the risk for bleeding, and the frailty and complexity of patients with underlying liver disease. Despite technological advances, it still constrains specific challenges such as rigid instruments, limited maneuverability, and two-dimensional vision. Nonetheless, minimally invasive liver surgery (MILS) has finally gained wide adoption for both malignant and benign hepatic diseases, thanks to growing positive evidence. Benefits of MILS include shorter hospital stays, faster recovery, and reduced postoperative morbidity and mortality, with comparable oncologic outcomes to open surgery [1,2,3]. However, despite the benefits, MILS approaches account for some limitations, particularly for certain liver resections considered technically challenging, such as those involving the posterosuperior segments of the liver [4].

The isolated resection of the caudate lobe represents a particularly demanding procedure due to its complex anatomical location and proximity to major vascular structures [5]. In particular, the caudate lobe usually has several veins that drain directly into the IVC, or portal branches coming directly from the left or the right portal vein [6,7]. Due to such difficulties, most of these resections were performed by an open approach until recently. Nonetheless, a recent meta-analysis showed that the laparoscopic approach may be associated with less intraoperative blood loss, less transfusion requirement, shorter hospital stay, and abbreviated operative times compared to an open approach [8].

Despite these findings, laparoscopic surgery has some limitations, such as the possibility of controlling intraoperative bleeding, the technical difficulty of suturing in vascular structures, and resecting tumors larger than 5 cm [6].

In this context, the use of a robotic surgical system may offer potential advantages, including enhanced three-dimensional view, better suture capability, abolishing the tremor, and facilitating the dissection of the hepatic hilum and the hepato-caval plane that could help when operating difficult areas of the liver [4,9]. Even in the case of, postero-superior lesions, robotic liver resections (RLR) have demonstrated improved postoperative outcomes when compared to the laparoscopic approach [4]. A meta-analysis of propensity score-matched studies from Giannone et al. reported similar perioperative results between the two approaches, with even reduced blood loss, lower blood transfusion rates, decreased conversion rates, and shorter operative times [10].

Despite the reported safety and feasibility of the RLR for posterosuperior segments, currently, the isolated robotic resection of the caudate lobe has not been fully explored. Existing reports are limited to small case series and a few comparative studies, lacking a consensus that addresses whether this type of resection is safe, effective, and comparable with the other approaches available [4].

The aim of this study was to systematically review and report the postoperative outcomes of isolated RLR of the caudate lobe.

## 2. Materials and Methods

### 2.1. Literature Search Strategy

This systematic review was conducted in accordance with the PRISMA (Preferred Reporting Items for Systematic Reviews and Meta-Analysis) guidelines [11]. A literature search in the Medline (Pubmed) and Scopus electronic databases up to 19 December 2024 was performed, with no language or publication status restriction. Additional potentially relevant studies were identified from the reference list of selected studies. The following search terms were used: (posterior OR postero* OR difficult OR caudate OR Spiegel OR segment one OR segment 1) AND (hepato OR hepat* OR liver) AND (resection OR hepatectomy) AND (minimally AND invasive OR robotic OR robot*).

**Figure 1:**
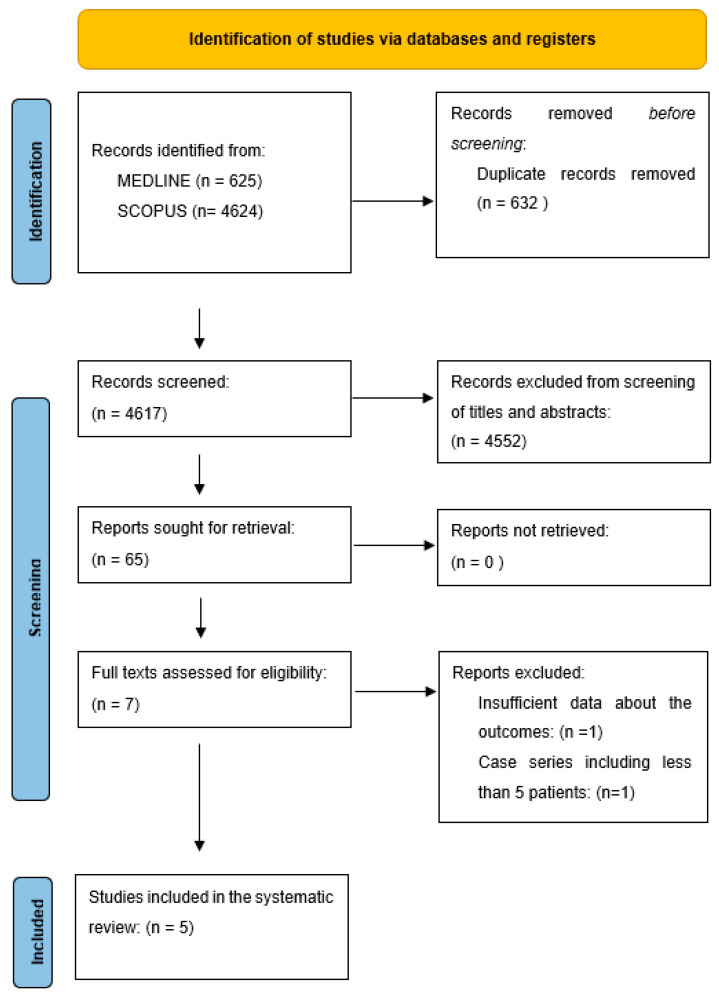
PRISMA flow diagram illustrating the study selection.

Two authors (GC and FG) independently screened the results of the electronic search at the title and abstract levels. The full text of the selected references was then retrieved for further analysis and data extraction. The PRISMA diagram for the selection of the studies is shown in Figure 1.

### 2.2. Study Selection

Two authors (GC and FG) independently screened the available articles, and the inclusion criteria were as follows: (i) studies investigating postoperative outcomes after isolated RLR for segment 1, and (ii) available data regarding intraoperative and postoperative outcomes. The exclusion criteria were as follows: (i) did not clearly report perioperative outcomes, (ii) include less than 5 patients, (iii) articles written in a language other than English, (iv) articles reporting outcomes of combined resections (caudate lobe + other segments), (v) combined procedures during the resection such as ablation, hand-assisted or hybrid techniques, and (vi) animal studies or (vii) duplicate studies.

### 2.3. Data Extraction

Two researchers (GC and FG) retrieved the data from the studies. In cases of discrepancies, a third researcher was involved (GDAM). The data obtained from the studies were first author’s name, publication year, title, journal, country, article type, number of patients, patient demographics (age, sex, body mass index (BMI), ASA Score, underlying cirrhosis, diagnosis, previous abdominal surgery and previous liver surgery), intraoperative outcomes (operative time, intraoperative blood loss, blood transfusions, need and cause of conversion, use and time of Pringle maneuver), postoperative outcomes (length of hospital stay, postoperative morbidity, major morbidity, need for reoperation, 30-day mortality, 90-day mortality, 30-day readmission rate, and R0 resection rate).

### 2.4. Statistical Analysis

Data were summarized and analyzed with descriptive statistics, based on their distribution. The global mean of variables from the studies was calculated as a weighted average of the group means based on the sample sizes. The global standard deviation was estimated using a pooled approach, accounting for the variance within each group and the sample sizes. For specific variables lacking direct standard deviations, estimates were derived from the means, assuming an asymmetric sample size distribution, as described by Luo et al. and Wan et al. [12,13].

The quality of studies included in this systematic review was scored by two researchers using the modified Newcastle Ottawa Scale (NOS) (with a score ranging from 0 to 9 points). The NOS is a review tool for evaluating the risk of bias in observational studies. The scale consists of four domains of risk of bias assessment: (i) selection bias; (ii) performance bias; (iii) detection bias; and (iv) information bias [14].

## 3. Results

### 3.1. Selected Studies

A total of seven studies met the inclusion criteria [15,16,17,18,19,20,21]. At the full-text review, two studies were excluded, due to insufficient patient information [18,20].

Five studies were included in the final review, three of them were case series and two cohort studies. Data from the included studies is shown in Table 1. One study reported 13 caudate lobe resections; however, in two patients, additional liver resections were performed, and four of them included combined resections with other abdominal organs. Thus, to minimize bias, only the seven patients who had an isolated resection of the caudate lobe were included in the review [17].

None of the included studies performed a comparative analysis between robotic caudate lobe resection and alternative techniques.

### 3.2. Patient Characteristics

A hundred and ten patients were included in the selected studies, with sixty (54.5%) patients identified as male. The mean age was 55.8 years, with ages between 27 and 75. ASA Risk Score was reported by three studies, with a median score of 2; 14 (20.6%) patients with ASA ≥ 3 were operated [15,16]. Previous abdominal or liver surgery was only reported by two studies, with 33 (54.1%) patients having a prior surgical record, and only 2 patients having a history of liver resection [15,21]. Twelve patients had an underlying hepatopathy, and only one patient was reported with a diagnosis of liver cirrhosis with a Child-Pugh A score [15,21]. Patients’ characteristics and preoperative data are shown in Table 2.

Sixty-two (56.3%) patients were operated due to a malignant tumor, while thirty-six (32.7%) of them were operated for a benign tumor. Twelve patients (10.9%) underwent surgery due to neoplastic lesions of the liver, but not specified. Among malignant tumors, three studies reported the histological types, with hepatocellular carcinoma being the most common type of malignant tumor 31 (50%), followed by 17 metastatic lesions (27.4%) [16,19]. Only one study included patients with intrahepatic cholangiocarcinoma, without reporting the exact number of cases of this diagnosis.

Tumor size varied substantially across studies. Marino et al. reported a mean size of 2.63 cm, referring to their inclusion criteria for tumors under 3 cm [15]. In contrast, Zhao et al. reported a mean size of 5.3 cm showing the general larger size of their series, similar to the median size reported by Jones et al. [14,18]. Sheng et al. reported a median size of 4 cm, with a maximum diameter reported as high as 5 cm, while these data were missing in the remaining studies [17]. Tumor characteristics are summarized in Table 2.

No data regarding lymph nodes or the use of neoadjuvant therapy were reported in the studies.

### 3.3. Intraoperative Outcomes

The mean operative time across the studies was 184.5 min, with reported times ranging from 70 to 522 min. Mean blood loss was 95.5 mL, and blood transfusions were required in 5 cases (5.6%). However, two studies did not report the transfusion rate [15,17]. No conversion to open surgery occurred.

Pringle maneuver was routinely used in 55 (50%) patients. Donisi et al. reported a median time of the Pringle maneuver of 30 min (ranging from 27 to 46) [21]. The rest of the studies did not report specific data regarding the time of application of this maneuver. Perioperative outcomes are shown in Table 3.

### 3.4. Postoperative Outcomes

Complications occurred in 24 (21.8%) patients. Only two patients presented a major complication (1.8%), while one of the articles, by Jones et al., did not report the rate of minor complications [15].

Liver-specific complications including postoperative hepatic failure were reported in two studies, without any incidence. Only one patient (0.9%) presented a bile leak requiring an abdominal drain punction. The rest of the studies did not assess the specific occurrence of these complications. Reoperation rate was reported by two studies, occurring in one patient due to a small bowel injury. Readmission occurred in two patients (1.8%).

The mean hospital stay was 4.2 days, with the longest hospital stay being reported in 19 days. Mortality at 30 days was null, as well as the 90-day mortality reported by two studies [15,16].

R0 Resection was achieved in 51 (46.4%) of the patients, not being reported in the rest.

### 3.5. Risk of Bias in Included Studies

The NOS was applied to score and classify the quality of these studies, as displayed in Supplementary Material Table S1.

## 4. Discussion

This systematic review was aimed to investigate the postoperative outcomes of RLR of the caudate lobe, showing safe and effective results in the selected cases. According to the authors’ opinion, the most important finding emerging from this study is the low number of studies available in the literature regarding this procedure, as well as the low quality of the evidence.

Liver surgery has evolved significantly in the past years, particularly with the adoption of minimally invasive approaches [22]. MILS was initially limited to specific selected cases, as stated in the first consensus conferences held in Louisville in 2008 and in Morioka in 2014 [23,24]. However, since the surgical experience continued to develop, indications for MILS have increased; such is the case for patients with liver cirrhosis and tumors in posterosuperior segments [25,26]. The implementation of robotic systems in liver surgery has provided numerous advantages, including reduced blood loss, shorter hospital stays, and less postoperative pain [22]. The key features of robotic surgery such as abolishing the surgeon’s tremor, amplifying the vision, and adding preciseness to the procedure, have opened the possibility of performing more complex procedures in liver surgery that were usually reserved for an open approach. Robotic surgery now has been proven to be safe in complex resections of tumors located in posterior segments, closed to vessels, and resection with a higher difficulty score [27]. The caudate lobe, also known as segment 1 in the Couinaud system, has always been considered technically challenging for resection. It comprises three portions: the Spiegel’s lobe, the caudate process, and the paracaval portion. Altogether, they are closely related to the vena cava posteriorly, the portal triad inferiorly the hepatic venous confluence superiorly [28]. Resection of the caudate lobe, either partial or complete, has been considered demanding even for experienced hepatobiliary surgeons. Similarly, the caudate lobe can be approached from the right or left of the hepatoduodenal ligament, anteriorly in a transhepatic approach or using a combined technique between the left and right accesses to the segment [21].

Recently, with the increasing use of advanced imaging techniques and the extension of resection criteria in many conditions of the liver, the need to perform an isolated surgery of the caudate lobe has become more frequent [29]. Open resection of the caudate lobe has traditionally been considered the preferred approach, mainly to allow better control of the vascular structures and avoid any potential injury. Performing an open resection requires a large abdominal incision conferring the risk of more postoperative pain, pulmonary complications, surgical site infection, reduced mobility, and increased length of hospital stay [21]. On the other hand, minimally invasive procedures have been shown to overcome some challenges in the resection of this segment [30]. Recent reports state that the laparoscopic approach can allow a caudal view enhancing the identification of vessels and the relationships of the segment with the surrounding structures [6,29].

Robotic resection of the caudate lobe not only provides better visualization of the segment but also offers better control and precision in the management of the adjacent vessels. The features of the robotic platform such as the stable camera and wider view field in narrow spaces, stable retraction, and exposure, and enhanced ability for suturing with the Endo-wrist system, grant the robotic approach a considerable advantage for resecting this segment [21]. Several considerations for the RLR of the caudate lobe include the use of intraoperative ultrasound and guidance tools, such as indocyanine green fluorescence. Both techniques are significantly enhanced by the robotic platform and have demonstrated considerable utility in liver surgery, irrespective of the surgical approach employed [21,31].

Currently, RLR of the caudate lobe has been reported only in small case series and case reports. Most of these studies addressed patients with tumors in difficult locations, size <3 cm, without lymph node metastasis or vascular involvement, identified as the best candidates when choosing the robotic approach [18,20]. Standardized indications, however, remain undefined.

In this review, two of the studies contraindicated the robotic approach when patients had tumors >3 cm, diagnosis of intrahepatic cholangiocarcinoma, suspected vascular invasion, positive lymph nodes, obesity (BMI > 35), an ASA score >3, or any contraindication for the pneumoperitoneum [16,19]. Interestingly, the other two studies included patients with a diagnosis of cholangiocarcinoma, suspected invasion to the Cava vein, with mean sizes of the resected tumors >3.8 cm. These studies show a minor operative time and blood loss when compared to the series of Marino et al. which included smaller-sized tumors [15,17]. This suggests that robotic surgery may be safe even in larger tumors with closed relations with the vessels.

In previous reports, laparoscopic resection of the caudate lobe has been shown to be superior to an open approach. In a recent systematic review including 221 patients, the mean operative time was 210 min, and blood loss was 173.6 mL. Results of this review showed that both the operative time of 184.5 min and blood loss (95.5 mL) of the robotic approach seemed to be more favorable [32]. It is important to notice that none of the included studies properly defined operative time. The longer operative times associated with robotic surgery have usually been considered a disadvantage. In a comparative analysis of different types of liver resections performed by open, laparoscopic, or robotic approaches, longer operative times were associated with a higher rate of complications and longer hospital stays, regardless of the technique. The robotic approach showed the lowest odds of complication compared to the other two vias, although there is a recognized bias in the patient selection [33]. It would be interesting to specifically compare RLR with LLR for isolated caudate lobe resection in the future.

Conversion to open surgery did not occur in any of the included patients, while the reported rate of conversion in laparoscopic surgery in other studies is as high as 3.1% [32]. Similar results have been reported also for laparoscopic and robotic resection of other difficult hepatic segments [34,35].

Overall postoperative complications in this review occurred in 21.8% of patients, while previous studies reported rates between 22 and 41% in open procedures [7,36]. Dorovinis et al. reported an overall complication rate of 16.28% for laparoscopic caudate lobectomy and 5.88% for major complications. In contrast, the rate of major complications in the robotic approach in this study was as low as 1.8% [32].

The primary indication for robotic caudate lobe resection was malignant tumors. Historically, minimally invasive techniques faced challenges in achieving adequate resection margins [35]. Current evidence of isolated resection of segment 1 mainly focuses on achieving safety and comparative postoperative results. Although little has been reported on long-term follow-up, in this review R0 was achieved for all the patients in whom this outcome was evaluated. Larger studies with longer follow-up periods are needed to determine the oncological safety of this approach.

This review is constrained by the small number of patients and the lack of evidence reporting RLR of the caudate lobe. The existing literature is limited by small sample sizes, heterogeneous data, and methodological variability among studies, as well as the lack of evidence in oncological outcomes and cost analysis of this approach.

Despite these limitations, this study reports the first experiences about the safety of RLR for S1, aiming to encourage further appropriate studies to confirm these promising results, as well as, ideally, to establish standardized indications, refine surgical techniques, and evaluate long-term oncological outcomes and feasibility of the robotic resection of the caudate lobe.

## 5. Conclusions

Few cohort studies investigating the outcomes of RLR for S1 are actually available in the literature. Robotic resection of the caudate lobe may be a safe technique in selected cases in experienced HPB centers. Larger studies are needed to identify the adequate selection criteria for patients who would benefit from this approach.

## Figures and Tables

**Table 1:** Data of the selected studies.

Author	Publication Year	Country	Type of Article
Marino et al. [16]	2018	Spain	Case Series
Zhao et al. [19]	2020	China	Cohort study
Sheng et al. [17]	2022	China	Case Series
Jones et al. [15]	2024	US	Cohort study
Donisi et al. [21]	2024	International	Case series

**Table 2:** Patient and tumor characteristics.

Author	Number of Patients, n	Sex (Male) n, (%)	Age (Years)	BMI (kg/m^2^)	ASA Risk Score	Malignant Tumor n (%)				Benign Tumor n, (%)	Tumor Size (cm)
						Metastatic	HCC	ICC	Other		
Marino et al. [16]	10	7 (70)	54.7 ± 10	27.5 *	2.1 ± 0.7	2 (20)	5 (50)	0 (0)	0 (0)	3 (30)	2.63 ± 0.53
Zhao et al. [19]	32	21 (65.63)	49.59 ± 14.58	24.61 ± 0.93	N.A.	1 (3.13)	17 (53.13)	0 (0)	1 (3.13) ^a^	13 (40.63)	5.28 ± 3.67
Sheng et al. [17]	7	2 (28.57)	42 ± 9.5	42 ± 9.5	N.A.	N.A.	N.A.	N.A.	1 (14.29) ^b^	6 (85.71)	4 ± 1.3
Jones et al. [15]	19	8 (42.11)	58 ± 16.3	29 ± 6.4	2.5 ± 0.51	N.A.	N.A.	N.A.	8 (42.11) ^b^, 11 (57.89) ^a^	N.A.	4.7 ± 3.03
Donisi et al. [21]	42	22 (52.4)	62 *	27.2 *	N.A.	19 (45.3)	9 (21.49)	0 (0)	0 (0)	10 (23.8)	N.A.

^a^ Other not specified neoplastic lesions. ^b^ Other not specified malignant lesions. * Insufficient data to estimate the standard deviation. N.A.: not available.

**Table 3:** Perioperative and oncological outcomes. N.A.: not available.

Author	Operative Time (min)	Blood Loss (mL)	Blood Transfusion n (%)	Conversion n (%)	Postoperative Morbidity n (%)	Major Postoperative Morbidity n (%)	30-Day Postoperative Mortality n (%)	Length of Hospital Stay (days)	Resection R0 n (%)	Overall Survival 1-Year n (%)
Marino et al. [16]	258 ± 93	136.7 ± 77.25	0 (0)	0 (0)	2 (20)	1 (10)	7 (0)	7.2 ± 2.25	N.A.	N.A.
Zhao et al. [19]	159.35 ± 57.85	194.34 ± 299.47	3 (9.38)	0 (0)	15 (46.88)	0 (0)	0 (0)	5.70 ± 3.09	32 (100)	32 (100)
Sheng et al. [17]	155.71 ± 45.4	42.14 ± 30.3	N.A.	0 (0)	0 (0)	0 (0)	0 (0)	3 ± 0.82	N.A.	N.A.
Jones et al. [15]	209 ± 153.5	72 ± 70.4	N.A.	0 (0)	N.A.	0 (0)	0 (0)	3 ± 1.9	19 (100)	19 (100)
Donisi et al. [21]	180 ± 36.4	30 ± 28.7	2 (4.8)	0 (0)	7 (16.7)	1 (2.4)	0 (0)	3 ± 0.57	N.A.	N.A.

## Supplementary Materials

The following supporting information can be downloaded at: https://www.mdpi.com/article/10.3390/medicina61010034/s1, Table S1: Modified Newcastle Ottawa Scale for the included studies.
