# Supplementary material for: Postoperative Outcomes After Robotic Liver Resection of Caudate Lobe: A Systematic Review
Source: Medicina (Kaunas). 2024 Dec 29;61(1):34. doi: 10.3390/medicina61010034 (PMC11767131; doi:10.3390/medicina61010034)
Supplement: Supplementary file 1 [file medicina-61-00034-s001.zip › medicina-3390219-supplementary.pdf]

Table S1: Modified Newcastle Ottawa Scale for the included studies.

Study	Selection	Sample Size	Postoperative Outcomes Definition *	Adjust for Cofounders	Postoperative Outcomes Assessment *
Marino et al.	Moderate	High	High	High	No description
Zhao et al.	Moderate	High	High	High	No description *
Sheng et al.	High	High	High	High	No description
Jones et al.	High	High	High	High	No description
Donisi et al.	High	High	High	High	No description

* Postoperative Outcomes: Postoperative mortality 30 and 90-days, overall postoperative morbidity, Major postoperative morbidity (Clavien-Dindo ≥ 3).
